# General practitioner attitudes to the care of people with epilepsy: an examination of clustering within practices and prediction of patient-rated quality of care

**DOI:** 10.1186/1471-2296-6-9

**Published:** 2005-03-01

**Authors:** Ajay K Thapar, Martin O Roland

**Affiliations:** 1 School of Psychology, Cardiff University, Tower Building, Park Place, Box 901, Cardiff, UK; 2 Taff Riverside Practice, Riverside Health Centre, Cardiff, UK; 3 National Primary Care Research and Development Centre, University of Manchester, Manchester, UK

## Abstract

**Background:**

There is wide variation in the quality of care provided by primary care practices to individuals with chronic illnesses. Individual doctor attitudes and interest have been demonstrated to influence patient outcomes in some instances. Given the trend towards larger practices and part-time working, continuity of care is likely to fall and thus practice-based rather than individual general practitioner attributes and attitudes are likely to become increasingly important. The aim in this paper was to examine the extent to which individual general practitioner (G.P.) attitudes to the care of people with epilepsy cluster within practices and predict patient-rated quality of care.

**Methods:**

The sample consisted of 1255 people with active epilepsy (a recent seizure or on anti-convulsant medication for epilepsy) and 199 GPs from 82 general practices. Measures of GP attitudes (a 17-item GP attitudes questionnaire) and patient-rated quality of epilepsy care were obtained. 1210 individuals completed initial questionnaires and 975 patients filled in final questionnaires one year later. Responses were achieved from 64 practices (83% of total) and 115 GPs (60% of total).

**Results:**

2 main factors were found to underlie GP attitudes to the care of people with epilepsy and these demonstrated clustering within practices "epilepsy viewed as a primary care responsibility" (Eigenvalue 3.98, intra-class correlation coefficient (ICC) 0.40), and "medication skills"(Eigenvalue 2.74, ICC 0.35). GP-rated scores on "epilepsy care being a primary care responsibility" were a significant predictor of patient-rated quality of GP care (p = 0.031). Other contributory factors were seizure frequency (p = 0.044), and patient-rated "shared decision making" (p = 0.022).

**Conclusion:**

Specific general practitioner attitudes to the care of people with epilepsy cluster within practices and are significantly associated with patient-rated quality of epilepsy care. It is important to take these findings into consideration when planning primary care interventions to ensure people with epilepsy receive the benefits of available medical and surgical expertise.

## Background

There is wide variation in the quality of care provided by primary care practices to individuals with chronic illnesses [1]. Individual doctor attitudes and interest have been demonstrated to influence patient outcomes in some instances [2,3] and it has been argued that specific attitudes are more predictive of behaviour than general attitudes[4]. For chronic diseases, in line with the distinction proposed by Katz [5], these doctor attitudes may be separated into perceptions of knowledge, skills and personal preferences. The importance of these specific doctor attitudes on patient outcomes is however largely unknown.

There is a marked trend to larger partnerships in primary care practices and more flexible working practices. It is likely, therefore, that continuity of care will continue to fall, and that patient experience of care of a particular condition will be based on contact with more than one general practitioner [6]. Thus practice-based rather than individual general practitioner (GP) attributes and attitudes are likely to become increasingly important. The extent to which GP attitudes to specific chronic conditions cluster within practices, is however currently unknown. There is evidence that where attitudes within a group are shared this enhances the influence of individual attitudes on behaviour [7]. Thus, on the basis of this observation, if attitudes are shared by general practitioners within practices, these group-based attitudes are then more likely to influence GP behaviour and the quality of care provided by the clinician.

In the next few years there is likely to be considerable reorganisation of the way in which epilepsy care within primary care is delivered, with GPs taking on a more active role in providing care. Information on how individual general practitioners view and value their role in providing epilepsy care is considered as important [8]. However, what may be more important is whether or not these views are shared within the practice and if these attitudes influence the quality of care provided by the practice. If this is the case, then taking GP attitudes in a given practice into account will be crucial in deciding how primary care services for people with epilepsy are best organised and improved.

In this paper, data from a completed community-based study on people with epilepsy are used to examine the following questions:

1. To what extent do individual general practitioner attitudes to the care of people with epilepsy cluster within practices?

2. Do general practitioner attitudes predict how people with epilepsy rate the quality of the general practitioner care of their epilepsy?

## Methods

General practitioners and adults with epilepsy taking part in an intervention study in Greater Manchester provided information for this study. The results of the intervention study (a prompt and reminder card for general practitioners to complete, held by patients or placed in their medical records and used opportunistically over the course of a year) have been reported [9]. No group differences in patient rated outcome measures were found for the intervention study [9].

Ethical approval was obtained in the 4 areas of Greater Manchester from where patients were approached. The patients who consented to participate in the study had their medical records examined to extract data on recording of clinical information about epilepsy and other markers of quality of care. Patients were also sent questionnaires for self-completion. These included both a generic quality of life measure- the EUROQOL 5D[10] and a disease specific quality of life and quality of care measure, the "Living with Epilepsy" questionnaire which has been psychometrically tested and shows good reliability and validity [11]. Self-rated seizure frequency (included in the "Living with Epilepsy" questionnaire) was based on the response to a question "How many epileptic attacks have you had in the past year" with the 3 response categories being "None", " Less than one a month" and "One or more a month".

General practitioners completed a 17-item GP epilepsy attitudes questionnaire at the end of the study. Responses to items such as "I feel comfortable changing the type of anti epileptic drugs in my patients" were scored using a Likert scale. The attitudes scale was developed and validated for a previous study and results reported in an earlier paper [12].

### Statistical analysis

Mean practice scores for each item on the GP attitudes questionnaire were computed and significant factors underlying the grouping identified (using eigenvalues >1.2 and the Scree test.). Clustering of responses on the attitudes questionnaire were examined using the intra-class correlation coefficient both for individual items as well as for a mean score of responses to items loading on each of the main factors. Finally linear regression analysis with the patient-rated quality of care provided by the practice as the dependent variable and GP attitudes and other patient derived measures (such as seizure frequency, age, gender, other long term illness) as independent variables was carried out (using aggregated GP attitude and patient scores). Intervention group was adjusted for. P values of 0.05 and 95% confidence intervals were used to assess significance.

## Results

1255 patients consented to participate in the study and 975 patients filled in final questionnaires. 199 GPs from 82 practices consented to participate in the study. Responses were obtained from 115 GPs (60% of total) from 64 practices (83% of total). 29 practices had a single respondent. These practices were excluded from the analysis of attitude clustering. In this study 54% of individuals were seizure-free in the previous year ("controlled" seizures) and 46% had reported a seizure in the previous year ("uncontrolled" seizures).

### Factor analysis and clustering of GP attitudes

Factor analysis with varimax rotation was undertaken on aggregate GP responses. Four factors had an eigenvalue of above 1.2. Three of these factors were selected after the scree test. Both the Kaiser-Meyer-Olkin measure of Sampling adequacy test (0.778) and Bartletts test for sphericity (Chi-square 413.7, Df 120, p < 0.0001) suggested that factor analysis was appropriate for this data set. Using guidelines for identifying significant factor loadings based on sample size from Hair et al.[13] a cut-off of 0.65 was used. Individual items within each factor were used to generate mean factor scores. These mean factor scores were normally distributed.

Responses to the 11 questions that were included in the first three factors were further examined. The aim was to detect if significant clustering of responses to these items occurred within practices. The average cluster size was 2.74.

The results of the factor analysis and clustering of attitudes for the two main factors are listed in Table 1. The other two factors were excluded. The first excluded factor was not clinically meaningful with only two disparate items loading on it ("epilepsy care straightforward", "epilepsy patients viewed as being well-informed"). The second excluded factor explained less than 10% of variance and only had one item loading on it ("self-perceived knowledge of epilepsy").

**Table 1:** Two main extracted factors, significant factor loadings and intraclass correlation coefficients (ICC) for general practitioner attitudes within practices

	Factor loading^1^	ICC^2^
**Factor 1: "Primary care responsibility"(Eigenvalue 3.98, 24.9% of variance explained)-mean scores**		**0.40**

"Not too time pressured to take on epilepsy care"	0.785	0.37**
"GP has primary responsibility for organising follow up care"	0.769	0.13
"Epilepsy care not too difficult to organise"	0.767	0.19*
"Epilepsy care not a specialist responsibility"	0.732	0.34**
"Epilepsy care should be based in general practice"	0.684	0.44**
"Annual structured review should be carried out in primary care"	0.657	0.10

**Factor 2: "Medication skills"(Eigenvalue 2.74, 17.1% of variance explained)**		**0.35**

"Comfortable adjusting dose of medication"	0.724	0.31**
"GP responsible for adjusting treatment if more fits"	0.718	0.25*
"Comfortable adjusting type of medication"	0.655	0.17

p < 0.05, ** p < 0.01

^1 ^(based on mean GP scores per practice)

^2 ^(based on individual GP scores in practices with >1 respondent)

### Do GP attitudes predict how patients rate the quality of GP care of their epilepsy?

Data from 60 practices where both patient and general practitioner data were available were used in the linear regression analysis. As the data were obtained at the end of an intervention study, the intervention group was also included as an independent variable. The results of this analysis are given in Table 2. Significant predictors of patient-rated quality of GP care were **patient **seizure frequency and **patient**-rated "shared decision making" and **GP**-rated score on "epilepsy care being a primary care responsibility" (Factor 1). Recording of clinical information about epilepsy was not a significant predictor of patient-rated quality of GP care.

**Table 2:** Linear regression analysis: GP and patient predictors of patient rated satisfaction with GP care of epilepsy

	Unstandardized Coefficient		Standardized Coefficient	t value	Sig.	95% Confidence intervals for B	
				
	B	Std Error	Beta			Lower bound	Upper bound
***Patient measures***							

Age	-.012	.006	-.19	-1.80	.079	-.025	.001
Gender	-.4	.258	-.19	-1.55	.128	-.921	.120
Long term health problems other than epilepsy	-.141	.255	-.05	-.55	.583	-.655	.373
Anxiety score	.051	.028	.24	1.80	.078	-.006	.108
Depression scores	-.048	.041	-.17	-1.16	.255	-.131	.036
Ease of talking to GP about epilepsy	.392	.348	.13	1.128	.266	-.309	1.109
GP takes views of epilepsy into account ("shared decision making")*	**.931**	**.390**	**.31**	**2.38**	**.022**	**.144**	**1.719**
Seizure frequency*	**.306**	**.148**	**.26**	**2.07**	**.044**	**.008**	**.604**
***GP measures***							
Epilepsy as primary care responsibility* (factor 1)	**.154**	**.069**	**.29**	**2.23**	**.031**	**.015**	**.294**
Medication skills (factor 2)	-.033	.059	-.07	-.564	.576	-.152	.086

Data adjusted for intervention group

r^2 ^= 0.635, adjusted r^2 ^= 0.525, standard error = 0.224

* p value <0.05

Some further bivariate analyses were also undertaken. Recording of clinical information about epilepsy by GPs was not significantly associated with the GP-rated score on epilepsy care being a primary care responsibility but was associated with seizure frequency.

## Discussion

In this study two main factors ("epilepsy viewed as a primary care responsibility" and "medication skills") were found to underlie GP attitudes to the care of people with epilepsy. Responses to questions constituting these factors demonstrated a high and significant level of clustering within practices. The main factor that accounted for the largest proportion of variance, general practitioner-rated "epilepsy viewed as a primary care responsibility", significantly predicted patient-rated quality of care. Patient-rated shared decision-making and seizure frequency were other significant predictors of patient-rated quality of GP epilepsy care. Recording of clinical information by GPs about epilepsy was not associated with GP attitudes to epilepsy care but was related to patient seizure frequency.

In this study general practitioner attitudes to the care of people with epilepsy were found to cluster within practices to a considerable extent. This has not previously been shown in the U.K. A recent Dutch study [14] showed that GPs working in the same partnership showed more resemblance in overall attitudes to patient care and behaviour than GPs not working in the same partnership and hypothesised that social processes in partnerships and local circumstances may be particularly relevant. The present study has quantified these intra-practice GP similarities in terms of attitudes to one specific chronic condition. Moreover the results of this study also demonstrate that certain general practitioner attitudes predict patient-rated quality of care provided by the practice. The high level of clustering of GP attitudes and the effect of these attitudes on patient-rated outcomes in terms of quality of care, may have important implications in determining the effectiveness of practice level interventions in primary care. These results suggest, firstly, that when planning educational interventions, changing GP attitudes within practices should also be a key aim and, secondly, to focus on changing attitudes for the practice as a whole rather than simply for individual general practitioners. In addition, for practice level intervention studies (especially those using patient-rated quality of care as an outcome measure) an estimate of clustering of doctor attitudes as well as estimates of clustering of patient responses when carrying out power calculations should be incorporated to avoid making a Type 1 error.

There is relatively little information of the relationship of GP attitudes to patient ratings of the quality of GP epilepsy care. Existing evidence suggests that GPs with a special interest in a particular condition improves outcomes [2]. The results of the present study extend these findings by highlighting the importance of specific attitudes (accepting a key role in management) rather than perceptions of specific skills (skills in medication management) in predicting patient rated quality of care.

The results of a multilevel analysis examining patient and doctor predictors of patient satisfaction from the Netherlands [15] suggested that most of the variance in "patient satisfaction" scores was at the patient level (age, morbidity and previous negative experience with the GP being the main predictors) with only 5–10% of the variance in "patient satisfaction" being at the doctor level. However in that Dutch study [15], specific GP attitudes were not included as predictors and the "patient satisfaction" score was a composite score incorporating measures of accessibility, availability, humaneness of the GP and information provision. Patient-centred communication skills are known to be associated with improved patient satisfaction [16] and our analysis indeed found that patient-rated shared decision making ("GP took my views into account") was another significant predictor.

Patient ratings of the quality of care do vary according to whether individuals have controlled or uncontrolled seizures. Individuals with controlled seizures rate the quality of care provided higher than individuals with uncontrolled seizures. However why the ratings of care provided are higher is not clear as individuals with controlled and uncontrolled epilepsy differ from each other in other characteristics that may influence quality ratings apart from seizure frequency (e.g. depression scores, social functioning).

At practice level GP attitudes are not related to mean practice patient seizure frequency. Although it is likely that individual GP attitudes will be influenced by whether an individual patient has controlled or uncontrolled seizures it is not possible to empirically demonstrate this relationship, as nearly all general practitioners will see a mix of individuals with "controlled" and "uncontrolled" seizures in a given year. Their attitudes to the care of people with epilepsy will be influenced by this spectrum of epilepsy severity (and often to a greater extent by other factors including significant events with individual patients).

In terms of limitations of the results, some practices did not consent to take part in this intervention study and not all GPs who participated completed questionnaires. However there were no significant differences between practices that participated and did not participate in terms of size, average deprivation or training status [9]. Moreover responses to the GP questionnaire were received from over 80% of practices that participated and 60% of the doctors that participated. Aggregate scores were used when doctor and patient views were analysed. This will reduce variability and may result in a loss of statistical power. However given that many different patient-doctor encounters are likely within a given year this approach was the most pragmatic. Although the results were obtained at the end of an intervention study that may have influenced attitudes one of the groups in the study was a control group and no significant differences in GP attitudes between groups was found. Furthermore, results on GP attitudes in the present study were very similar to those found in a previous survey using this scale [12].

## Conclusion

Specific general practitioner attitudes to the care of people with epilepsy are significantly associated with patient-rated quality of epilepsy care and cluster within practices. It is important to take these findings into consideration when planning interventions and services. General practitioners need to have good knowledge and skills in the management of epilepsy and should be aware of and utilise current guidelines for good clinical epilepsy care [17-19] to fully utilize medical and surgical expertise in managing epilepsy. Recognising and addressing general practitioner attitudes to the care of people with epilepsy may be important in ensuring these goals of good epilepsy care are met.

## Competing interests

The author(s) declare that they have no competing interests.

## Authors' contributions

AT designed and ran the study, undertook the analysis and wrote the manuscript. MR was involved in the design and running of the study and edited the paper.

## Pre-publication history

The pre-publication history for this paper can be accessed here:


